# Impact of Oxidative Stress on Exercising Skeletal Muscle

**DOI:** 10.3390/biom5020356

**Published:** 2015-04-10

**Authors:** Peter Steinbacher, Peter Eckl

**Affiliations:** Department of Cell Biology, University of Salzburg, A-5020 Salzburg, Austria; E-Mail: peter.eckl@sbg.ac.at

**Keywords:** skeletal muscle, ROS, exercise, mitochondria, force generation, antioxidants, PGC-1α

## Abstract

It is well established that muscle contractions during exercise lead to elevated levels of reactive oxygen species (ROS) in skeletal muscle. These highly reactive molecules have many deleterious effects, such as a reduction of force generation and increased muscle atrophy. Since the discovery of exercise-induced oxidative stress several decades ago, evidence has accumulated that ROS produced during exercise also have positive effects by influencing cellular processes that lead to increased expression of antioxidants. These molecules are particularly elevated in regularly exercising muscle to prevent the negative effects of ROS by neutralizing the free radicals. In addition, ROS also seem to be involved in the exercise-induced adaptation of the muscle phenotype. This review provides an overview of the evidences to date on the effects of ROS in exercising muscle. These aspects include the sources of ROS, their positive and negative cellular effects, the role of antioxidants, and the present evidence on ROS-dependent adaptations of muscle cells in response to physical exercise.

## 1. Introduction

Skeletal muscle is a highly specialized tissue with excellent plasticity in response to external stimuli such as exercise and training. The repetitive muscle contractions conducted during endurance training lead to a variety of phenotypic and physiological responses. These responses include activation of mitochondrial biogenesis, fiber type transformation and angiogenesis. Together, they increase the muscle’s capacity of aerobic metabolism and its resistance to fatigue. High muscle activity also involves a strong increase in reactive oxygen species (ROS) production. These unstable molecules and ions contain oxygen and are extremely reactive due to an unpaired electron. Among these oxygen intermediates are the free radicals superoxide, peroxide and the hydroxyl radicals and other highly reactive oxidants, such as singlet oxygen and hypochlorous acid. They promote oxidation reactions with other molecules, such as proteins, lipids and DNA and can thus be highly detrimental. However, recent research has demonstrated that ROS also have a beneficial role in promoting the adaptive responses of muscle to training.

More than three decades ago it was established that muscle activity leads to an increase in ROS production and concentration of free radicals [1,2]. Since then numerous investigations in rodents and humans have confirmed these early observations. Thus, it is generally accepted that single bouts of aerobic or anaerobic exercise, as well as chronic exercise promote the generation of ROS (summarized in the reviews [3,4]; and more recently e.g., [5,6,7]).

The great interest in this topic also stems from data that show that ROS levels are increased in subjects with aging-related sarcopenia, cardiac reperfusion injuries or muscular diseases, *i.e.*, muscle dystrophies. Thus, it was assumed that exercise-induced ROS are potentially detrimental to muscle function and lead to muscle fatigue and muscle atrophy. Hence, many investigations focused on ways to prevent ROS production and accumulation and subsequent oxidative damage during and following physical exercise.

## 2. Sources of ROS in Muscle

**Figure 1:**
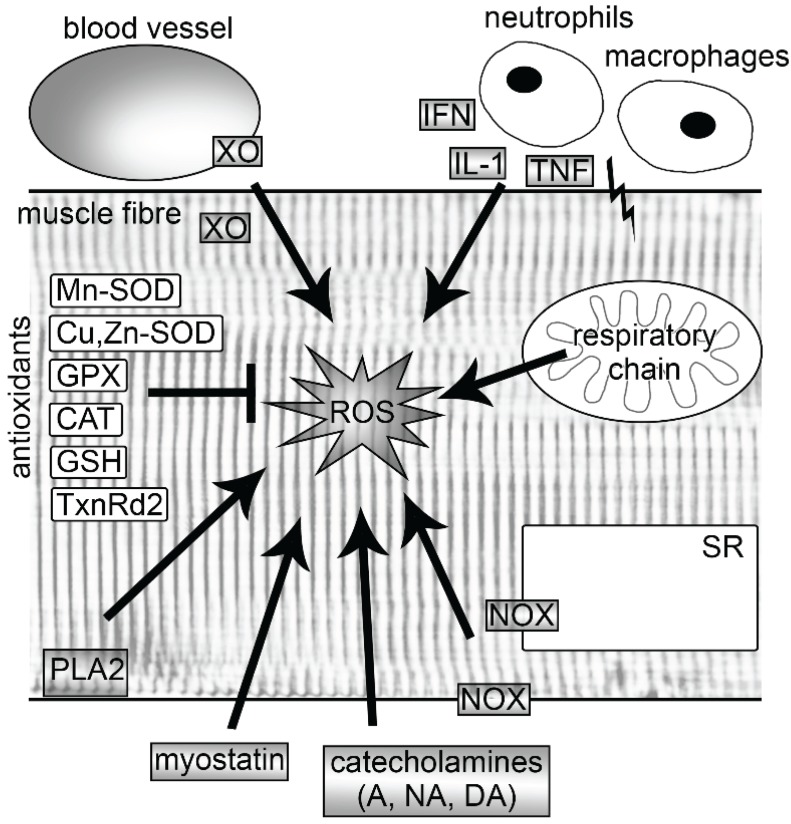
Sources of reactive oxygen species (ROS) and endogenous antioxidants in skeletal muscle fibers. Following exercise, ROS are produced endogenously by mitochondria, NOXs, PLA2 and XO. In addition, exercise increases ROS production also in activated neutrophils and macrophages, endothelia of blood vessels and by catecholamines. Regular exercise leads to an increase of endogenous antioxidants, which are able to neutralize free radicals. A, adrenaline; CAT, catalase; Cu, Zn-SOD, copper-zinc superoxide dismutase; DA, dopamine; GPX, glutathione peroxidase; GSH, glutathione; IFN, interferon γ; IL-1, interleukin-1; Mn-SOD, manganese superoxide dismutase; NA, noradrenaline; NOX, nicotinamide adenine dinucleotide phosphate oxidase; PLA2, phospholipase A2; SR, sarcoplasmic reticulum; TNF, tumor necrosis factor; TxnRd2, thioredoxin reductase 2; XO, xanthine oxidase.

It has consistently been shown that muscle activity leads to a strong increase in ROS production [8]. However, there is a large debate about the sources and the extent of ROS that these sources produce. Several potential producers of ROS have been identified in muscle cells which are likely to be activated by different stimuli. Among these are mitochondria, nicotinamide adenine dinucleotide phosphate (NADPH) oxidases (NOXs), phospholipase A2 (PLA2), xanthine oxidase (XO) and lipoxygenases (Figure 1). Some of these are discussed in more detail below. In addition to these intracellular sources, ROS has been shown to be produced from non-muscle sources. Strenuous exercise can elicit muscle injuries, which then lead to the activation of the neutrophils and macrophages via interferon-γ (IFN-γ), interleukin-1 (IL-1) and tumor necrosis factor (TNF) (for more detailed information see reviews [9,10]). These immune cells excessively produce ROS (oxidative burst), which is a central component of neutrophil defense mechanism. In addition, the exercise-induced increase of catecholamines (adrenaline, noradrenaline, dopamine) also play a role in the generation of ROS [11], as well as ROS derived from endothelium [12] (Figure 1).

### 2.1. Mitochondria

For a long period of time, mitochondria were regarded as the main producer of cellular ROS with an estimated superoxide production rate of approximately 1%–4% of total mitochondrial O_2_ consumption (see reviews [8,13,14]). More recent data demonstrate that the production of ROS in mitochondria is by an order of magnitude smaller than originally expected and is approximately 0.15% [15]. Mitochondria are thought to produce ROS by a leak of single electrons in the respiratory chain in the mitochondrial inner membrane of the contracting muscle cells. Ten different sites of superoxide/H_2_O_2_ generation have been found as yet in mammalian mitochondria [16,17]. Superoxide production mainly occurs from complexes I (NADH dehydrogenase) and III (coenzyme Q and cytochrome C oxidoreductase) of the electron transport chain [18,19]. New findings also identify complex II (succinate dehydrogenase) as a major source of superoxide production [16]. Using isolated mitochondria, the contribution of each site to total H_2_O_2_ production has recently been quantified and shown to strongly depend on the substrate being oxidized [20]. At rest, H_2_O_2_ was predominantly produced from the quinol site in complex I (site I_Q_) and flavin site in complex II (site II_F_), followed by sites I_F_ and III_Qo_. Under conditions that mimic mild and intense aerobic exercise, total production is much less and the low capacity site I_F_ dominates [20].

### 2.2. NADPH Oxidases

NADPH oxidases (NOXs) are flavoprotein enzymes that are activated by calcium, free fatty acids, protein-protein interactions and posttranslational modifications and use NADPH as electron donors [21,22]. They are transmembrane proteins in the transverse tubules and the sarcoplasmic reticulum and transport electrons across biological membranes to reduce oxygen to superoxide or H_2_O_2_ [21,22]. It was shown that NOX family members contribute to cytosolic superoxide production in skeletal muscle both at rest and during contractile activity to a larger extent than mitochondria [23,24,25]. ROS generated by NOXs activates ryanodine receptors (RyR), which leads to an intracellular Ca^2+^ release [26,27,28]. More recently, it was found that insulin induces ROS generation through NOX activation and that this ROS increase is required for the intracellular Ca^2+^ rise mediated by inositol triphosphate (IP_3_) receptors [29].

### 2.3. Xanthine Oxidase

Xanthine oxidase (XO) is a cytosolic molybdoflavoenzyme that is recognized as a key enzyme in purine catabolism in which it catalyzes the hydroxylation of hypoxanthine to xanthine and of xanthine to uric acid [30]. In muscle, XO is present in the cytosol but also in the associated endothelial cells [8]. Upon contraction, XO activity is significantly increased and leads to increased lipid peroxidation, protein oxidation, muscle damage and edema [31]. During intense exercise in which large amounts of ATP are consumed, hypoxanthine and xanthine levels are rising and serve as substrates for XO to generate ROS [32]. Interestingly, ROS generated by XO appears to be involved in the regulation of exercise-induced mitochondrial biogenesis via peroxisome proliferator-activated receptor-γ coactivator-1α (PGC-1α) [33].

### 2.4. Myostatin

Recently, it was demonstrated that myostatin, a blocker of muscle differentiation, is capable of signaling ROS production via canonical Smad3, nuclear factor (NF)-κB and TNF-α in muscle cells [34]. In the absence of Smad3, myostatin induces ROS production through the activation of p38 and ERK mitogen-activated protein kinase (MAPK) pathways mediated via TNF- α, IL-6, NOX and XO [35].

### 2.5. Phospholipase A2

Enzymes of the phospholipase A2 (PLA2) family also contribute to intra- and extracellular ROS increase during muscle contraction. They cleave arachidonic acid from phospholipids in the plasma membrane, sarcoplasmic reticulum or mitochondrial membranes. Arachidonic acid is an important lipid-signaling molecule and is a substrate for lipoxygenases for the production of ROS [36]. In addition, the cytosolic PLA2 enzyme has been demonstrated to increase ROS by stimulating NOXs [37]. Human muscle is known to contain approximately 15 different PLA2 isoforms that are either Ca^2+^-sensitive or Ca^2+^-insensitive [38]. The Ca^2+^-independent and dependent enzymes are supposed to produce ROS under resting and activity conditions, respectively [39].

## 3. Effects of ROS on Force Generation and Muscle Atrophy

**Figure 2:**
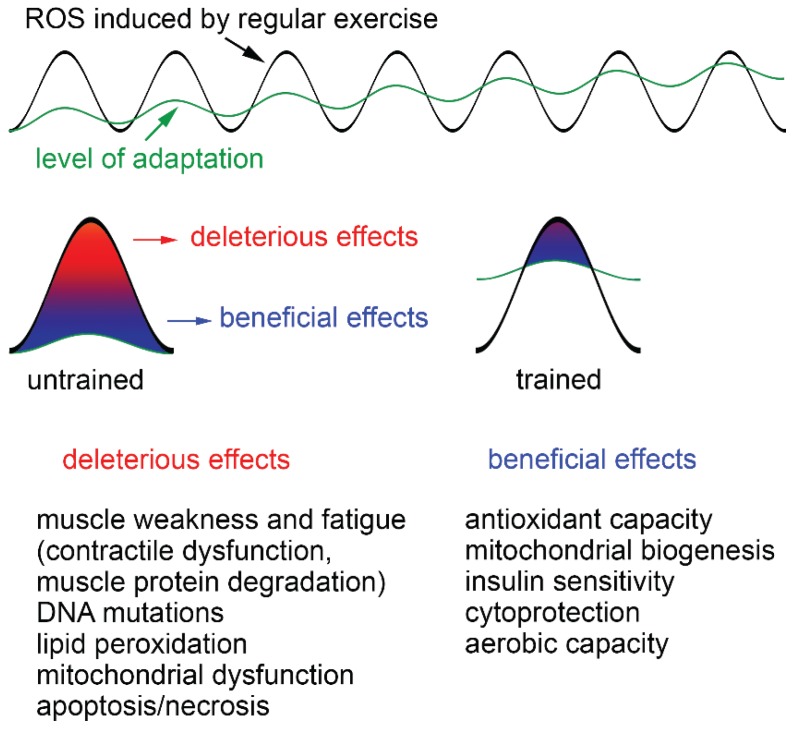
Deleterious and beneficial effects of exercise-induced ROS increase. Exercise produces ROS and whether they are beneficial or detrimental to health is dependent upon the ROS concentration, duration of ROS exposure and training status of the individual. A single bout of exhaustive exercise leads to strong increases of ROS, which cannot be buffered by endogenous antioxidants, particularly in untrained individuals. This results in severe oxidative damage, including muscle weakness and fatigue, DNA mutations, lipid peroxidation, mitochondrial dysfunction and apoptosis/necrosis. Trained persons have a higher level of adaptation and less health risks. ROS produced during regular exercise continuously increase the level of adaptation by improving antioxidant capacity, mitochondrial biogenesis, insulin sensitivity, cytoprotection and aerobic capacity of skeletal muscle.

In unfatigued muscle, intracellular ROS appear to be essential for normal force generation. Low-level ROS supplementation even increases force production [40]. A stronger increase of ROS due to intense exercise leads to a variety of adaptations of the muscle cells. Dependent upon the ROS concentration, duration of ROS exposure and training status of the individual, ROS can have beneficial and detrimental effects (Figure 2). Thus, a single bout of exhaustive exercise has been shown to cause oxidative damage in untrained persons while in trained subjects, no such effects are observed due to an increased resistance of such persons to oxidative stress [41]. Strong increases in ROS after strenuous exercise, aging and/or disease (e.g., chronic heart failure, COPD, cancer) can cause contractile dysfunction and muscle atrophy, which both promote muscle weakness and fatigue [3,42].

### 3.1. Contractile Dysfunction

Contractile dysfunction may result from oxidative modifications of a variety of proteins in diverse intracellular components [43,44]. However, our understanding of the processes involved is still limited and many data are equivocal. In the sarcoplasmic reticulum, the ryanodine receptor (RyR), which is the Ca^2+^ release channel, was shown to be oxidized by ROS and it was hypothesized to contribute to muscle fatigue [26,27,28]. However, other work demonstrated that this oxidation resulted only in increased Ca^2+^-induced Ca^2+^ release, whereas the Ca^2+^ release triggered by action potentials was not affected [45,46]. From this it was inferred that ROS-mediated effects on Ca^2+^ release in the sarcoplasmic reticulum are unlikely to contribute to muscle fatigue. This goes in line with recent findings that demonstrate that the mitochondrial antioxidant SS-31 restored the decrease in sarcoplasmic reticulum Ca^2+^ release while force recovery was not improved [47].

Similar uncertainty surrounds the effect of ROS on the force generating myofilaments. Initial data have shown that brief exposure to low concentrations of H_2_O_2_ increased force by 27%, while a longer exposure results in force decline [48]. By contrast, a short exposure of skinned fibers to 10 mM H_2_O_2_ had no effect on maximum force [45], while longer exposure to 50 mM H_2_O_2_ inhibited contractility [49]. Although the exact mechanisms are unknown, it is generally assumed that changes in force generation are the result of changes in the myofibrillar Ca^2+^ sensitivity [8,44,47]. In this regard, it was most recently suggested that troponin I, which is involved in sensing the intracellular Ca^2+^ levels, is a target of ROS. Oxidized cysteine residues of troponin I can react with the antioxidant glutathione, which helps protect the molecule from oxidative stress and make the contractile apparatus much more sensitive to Ca^2+^ [50]. However, it must be mentioned that ROS may also lead to changes in the contractile proteins. In this regard, it was demonstrated that H_2_O_2_ is able to modify the S1 fragment in the myosin head, which then leads to a restriction of the myosin-actin dynamics in the presence of ATP [49].

### 3.2. Muscle Atrophy

Besides their effects on the contractile kinetics, ROS are also able to modulate various signaling pathways, such as calcium, protein tyrosine kinases and phosphatases, serine/threonine kinases, and phospholipases [51]. This then leads to changes in gene expression, cell function, metabolism or cell damage. Chronic oxidative stress is associated with an increase in protein loss and muscle atrophy. High ROS levels cause a sustained activation of NF-κB and of FoxO which then activate two muscle-specific E3 ubiquitin ligases, atrogin-1 or muscle atrophy F-box (MAFbx) and muscle RING (Really Interesting New Gene)-finger protein 1 (MuRF-1) [52]. MAFbx and MuRF-1 then degrade various proteins, such as titin, nebulin, troponin, myosin-binding protein C, myosin light chains 1 and 2 and myosin heavy chain [53,54]. Recently, it was demonstrated that excessive oxidative stress also enhances the transcription factor C/EBP homology protein (CHOP). This transcription factor also enhances expression of MuRF1, which again results in increased protein degradation [35].

## 4. Antioxidants in Muscle

### 4.1. Enzymatic and Nonenzymatic Antioxidants

Muscle activity increases ROS but simultaneously also the body’s antioxidant defense system. These molecules are able to neutralize free radicals by accepting the unpaired electron and thereby inhibit the oxidation of other molecules. Depending on the oxygen consumption rate, cells constitutively express different levels of antioxidant enzymes, including mitochondrial antioxidant manganese superoxide dismutase (Mn-SOD, SOD2), cytosolic copper-zinc superoxide dismutase (Cu, Zn-SOD, SOD1), glutathione peroxidase (GPX) and catalase (CAT), and the nonenzymatic antioxidant glutathione (GSH) [55] (Figure 1).

GSH is the most abundant nonprotein thiol in cells with intracellular concentrations of 1–15 mM [56]. It plays a major role in the detoxification of electrophilic xenobiotics, such as chemical carcinogens, environmental pollutants, and the inactivation of endogenous α,β-unsaturated aldehydes, quinones, epoxides, and hydroperoxides, which are formed as secondary metabolites during oxidative stress via members of the glutathione transferase family [57]. It also protects from oxidative stress by reducing hydrogen peroxide and organic peroxides levels via a reaction catalyzed by GSH peroxidase thus keeping the intracellular environment in the reduced state [55,58]. In addition, GSH is a substrate for dehydroascorbate reductase enabling the recycling of ascorbic acid, and it is a scavenger of hydroxyl radicals and singlet oxygen [59].

The aforementioned enzymatic reactions lead to the oxidation of GSH to glutathione disulfide (GSSG). This molecule can inactivate a number of enzymes by reacting with protein thiols leading to the formation of mixed disulfides (e.g., [60]). To avoid damage to intracellular constituents, GSSG is efficiently reduced to GSH by glutathione reductase (GR) utilizing NADPH. This action of GR is also very important during and after exercise in which a substantial amount of GSH is oxidized due to the elevated ROS levels to keep the GSH/GSSG ratio constant thereby maintaining homeostasis. Furthermore, exercising skeletal muscle appears to increase GSH import from plasma [61,62], and liver can synthesize GSH *de novo* and supply it [58]. But also increased muscle glutathione synthetase activities have been observed after treadmill training [63]. These exercise responses are tissue- and fiber-specific [64].

The antioxidant enzymes SOD, CAT and GPX are the primary defense against ROS generated during exercise and increase in response to exercise [65,66]. Recent work identified thioredoxin reductase-2 (TxnRd2) as another key player to decrease the exercise-induced content of mitochondrial H_2_O_2_ in skeletal muscle [67]. The same authors have shown that TxnRd2 is also able to control mitochondrial H_2_O_2_ levels after a high-fat, high-sucrose diet in the heart but not in skeletal muscle. Antioxidant enzyme levels vary considerably with respect to muscle fiber types, *i.e.*, type I muscle fibers possess higher activity of all antioxidant enzymes than the type IIA and type IIB fibers [68].

### 4.2. Adaptive Responses to Exercise

In general, it was found that there is an exercise-induced increase in antioxidant protein levels and antioxidant activity. Thus, endurance training in rats leads to an increase in Mn-SOD, GPX and CAT, while the data on Cu, Zn-SOD are somewhat less clear [69,70,71,72,73]. Note that from the above studies, it is likely that upregulation of these antioxidants is muscle- and/or fiber type-specific. Many studies have shown that even an acute bout of exercise increases SOD activity in skeletal muscle ([74,75,76,77]; for review see [55]), and it has further been shown that Cu, Zn-SOD and Mn-SOD contents are increased. While the Cu, Zn-SOD enzyme activity gradually returns to resting levels within three days, Mn-SOD activity and protein content continues to increase in the post-exercise period [78]. GPX activity after acute exercise on the other hand appears to depend on the muscle type, *i.e.*, GPX activity was increased a day after an acute bout of treadmill running to exhaustion in rat soleus but not tibialis muscle [78], and CAT activity appears not to be altered by acute exercise [65]. 

ROS generated by acute exercise can lead to increased lipid peroxidation as measured by the formation of malondialdehyde [79]. Interestingly, this effect was only found in liver and fast skeletal muscle in the sedentary group, whereas the endurance-trained group did not show increases in lipid peroxidation after exercise. Lipid peroxidation generates a vast number of oxidative lipid breakdown products for which more or less specific tests are available (for review see [59]. Some investigators determined plasma isoprostane levels in athletes performing either a 50 km ultramarathon [80] or exercise for 2.5 h on a treadmill [81]. Peak levels of isoprostanes were found directly post-exercise, followed by a return to baseline within one day or one hour, respectively. Other investigators found increased levels of pentane in the breath after exercise [1], increased levels of lipid hydroperoxides [82], conjugated dienes [83] and oxidative DNA damage as measured by 8-hydroxydeoxyguanosine [84] or single cell gel electrophoresis [85]. The latter investigation gave results similar to those of Alessio and Goldfarb [79], namely a significant reduction of oxidative DNA damage in the trained compared to the untrained group indicating that exercise training causes an adaptive response to elevated oxidative stress by increased antioxidant enzyme activity. Powers *et al.* [86] studied the influence of training and observed significantly increased SOD activity in the soleus following exercise up to 60 min/d. Conversely, training induced significant increases in GPX activity in slow gastrocnemius only, and the magnitude of the GPX increase was directly related to exercise duration but relatively independent of intensity. However, CAT activity was not increased in any muscle with training. In addition, Radák *et al.* [87] observed decreased DNA damage and increased DNA repair levels as well as resistance against oxidative stress of proteins in aged rat skeletal muscle upon training. The obviously paradoxical situation that increased exercise-induced oxidative stress causes beneficial effects is interpreted in terms of hormesis—beneficial effects of potentially harmful agents—which apart from providing an adaptation to the damaging agent provide also systemic beneficial effects, including improved physiological function, decreased incidence of disease and a higher quality of life [88]. However, the beneficial effects appear to depend upon the duration of the exercise. While a single bout of exercise is suggested to lead to a limited adaptive response, regular exercise appears to gradually increase the level of adaptation by the repeated activation of antioxidant genes and proteins [32]. These authors hypothesize that it is the increased ROS level that is the important stimulus for the muscle cells to adapt to chronic exercise. The improved capabilities to decrease ROS may then provide a better protection from ROS during subsequent trainings but also attenuate the aging process and promote health with increased functional capacities [32]. Therefore, exercise is very similar to the adaptive ischemic preconditioning response [89]. Restoration of perfusion to ischemic organs results in increased ROS levels that can lead to tissue damage, myocardial infarction and stroke. However, such deleterious effects can be avoided by short intermittent bouts of reperfusion in which the transiently elevated ROS levels are important mediators of a cardioprotective response [89,90].

Important mediators of the adaptive responses are the adenosine monophosphate-activated protein kinase (AMPK), the transcription factors NF-κB, together with p38 MAPK, and members of the FoxO transcription factor family [91,92,93]. At low ROS levels, they promote adaptation by increasing gene expression of antioxidant enzymes, such as Mn-SOD and Cu, Zn-SOD, CAT and GPX1 [94]. High antioxidant capacities then diminish the deleterious effects of subsequent increases in ROS [95]. In addition, products of radical reactions are also suggested to be the mediator of this adaptation. Of special interest in this context are lipid peroxidation products, in particular 4-hydroxynonenal (HNE), which has been shown to both induce DNA damage [96] but also to be involved in the regulation of cell proliferation and growth as well as necrotic or apoptotic cell death by its marked ability to modulate several major pathways of cell signaling and, consequently, gene expression (for review see [97]). With respect to antioxidant gene expression it has been shown to be one of the most effective activators of nuclear factor erythroid-derived 2-like 2 (Nrf2) [98] which on stimulation dissociates from its cytoplasmic inhibitor Keap1, translocates to the nucleus and transactivates antioxidant-responsive elements (ARE)-dependent genes [99]. In addition, HNE has been demonstrated to cause mitochondrial uncoupling and thus protection from ROS specifically via the induction of the uncoupling proteins UCP1, UCP2 and UCP3 and the adenine nucleotide translocase [100].

### 4.3. Exogenous Antioxidants and Exercise

Apart from the endogenous antioxidants, which are obviously regulated by exercise, exogenous antioxidants such as vitamin C, E, and carotenoids are taken up with the food or are used as dietary supplements. The question therefore arises whether such supplements can be considered beneficial during exercise. To address this question, Ristow *et al.* [73] investigated the effects of a diet supplemented with vitamin C and E on exercise-induced insulin sensitivity as measured by glucose infusion rates during a hyperinsulinemic, euglycemic clamp in previously untrained and pre-trained healthy young men. Interestingly, exercise was found to increase parameters of insulin sensitivity (including adiponectin) only in the absence of antioxidants in both previously untrained and pretrained individuals. This was paralleled by increased expression of ROS-sensitive transcriptional regulators of insulin sensitivity and ROS defense capacity, peroxisome proliferator-activated receptor γ (PPARγ) and PPARγ coactivators PGC-1α and PGC-1β only in the absence of antioxidants. Molecular mediators of endogenous ROS defense (Mn-SOD, Cu, Zn-SOD and GPX) were also induced by exercise, and this effect was again blocked by antioxidant supplementation. The authors concluded that exercise-induced oxidative stress ameliorates insulin resistance and causes an adaptive response promoting endogenous antioxidant defense capacity and that supplementation with antioxidants may preclude these health-promoting effects of exercise in humans. It was demonstrated that exercise causes an activation of mitogen-activated protein kinases (MAPKs: p38, ERK 1 and ERK 2), which in turn activates nuclear factor κB (NF-κB) in rat gastrocnemius muscle and consequently the expression of important enzymes associated with defense against ROS (SOD) and adaptation to exercise—endothelial nitric oxide synthase (eNOS) and inducible nitric oxide synthase (iNOS) [101,102,103]. The expression of these enzymes can be inhibited by allopurinol, an inhibitor of XO indicating also that the prevention of ROS formation causes an inhibition of an adaptive response. The authors therefore conclude that in all likelihood, antioxidant supplements should not be recommended before training as they interfere with muscle cell adaptation. Thus, physical exercise is considered a double-edged sword: when practiced strenuously it causes oxidative stress and cell damage; in this case application of antioxidants may be helpful. But when practiced in moderation, it increases the expression of antioxidant enzymes and thus should be considered an antioxidant [101,103]. Supportive evidence for this assumption comes from studies on physical overtraining. Margonis *et al.* [104] examined the responses of oxidative stress biomarkers to a resistance training protocol of progressively increased and decreased volume/intensity in male test persons and observed significantly increased levels of urinary isoprostanes (7-fold), serum levels of thiobarbituric acid reactive substances (TBARS), protein carbonyls, CAT, GPX, and GSSG and significantly decreased levels of GSH, the GSH/GSSG ratio, and total antioxidant capacity in blood serum of over-trained individuals. Similarly, Palazzetti *et al.* [105] investigated the effects of overloaded training (OT) with athletes exercising for a duathlon before and after a four week OT and found that at rest conditions, OT induced an increased plasma GPX activity and a decreased plasma total antioxidant status, while OT resulted in higher exercise-induced variations of blood GSH/GSSG ratios, TBARS levels and decreased total antioxidant status in exercise conditions indicating that OT could compromise the antioxidant defense mechanisms. By comparing the oxidative stress response in control athletes and athletes with overtraining syndrome Tanskanen *et al.* [106] were further able to show that exercise to exhaustion led to an increase in oxygen radical absorbance (antioxidant) capacity and malondialdehyde in the controls but not in the over-trained athletes. Instead, over-trained athletes showed negative correlations between oxygen radical absorbance capacity at rest and protein carbonyls after exhaustive exercise indicating that increased oxidative stress may play a role in the pathophysiology of overtraining syndrome. Although these observations are not yet conclusive they indicate that adaptation to exercise is limited and that its protective effect can be exceeded leading to oxidative stress that cannot be dealt with by the endogenous antioxidant system. Whether it is helpful to apply exogenous antioxidants under such conditions as suggested still has to be elucidated.

## 5. Training-Induced Muscular Adaptation, PGC-1α and ROS

In addition to the above-described effects of exercise on contents and activities of antioxidant enzymes, regularly performed exercise in the form of endurance training leads to well described adaptations of the cardiovascular and muscular system. Important responses at the intramyocellular level include increases in size and number of mitochondria as well as such in the activities of oxidative enzymes [107,108,109]. In support of the increased oxidation of fatty acids, the content of intramyocellular lipid is also elevated [110]. Endurance exercise is also known to improve insulin sensitivity and muscular glucose uptake [108,111]. Recent research has demonstrated that ROS also have a beneficial role in promoting these adaptive responses of muscle to training.

### 5.1. Role of PGC-1α in Exercise

In rodents and humans, it has been demonstrated that peroxisome proliferator-activated receptor gamma coactivator-1 alpha (PGC-1α) is a key regulator of the exercise-induced changes of muscle fibers towards a slow phenotype, as well as in the protection from muscle atrophy [108,112,113]. Several studies have shown that PGC-1α is upregulated after high-intensity training [114,115,116,117,118]. Activation of PGC-1α is likely to occur by phosphorylation of the PGC-1α protein by p38 MAPK together with NF-κB [119], both of which are known to be activated by ROS [91,92]. PGC-1α has been demonstrated to regulate lipid and carbohydrate metabolism, and to improve the oxidative capacity of the muscle fibers by increasing the amount and activity of mitochondria through upregulation of nuclear respiratory factors (NRF-1, 2) and mitochondrial transcription factor A (TFAM) [120,121]. Furthermore, PGC-1α regulates genes involved in the determination of muscle fiber type. Overexpression of PGC-1α increases the proportion of oxidative type I fibers [122] while PGC-1α knock-out (KO) mice exhibit a shift from oxidative type I and IIA toward glycolytic type IID/X and IIB fibers [123]. This regulatory diversity of PGC-1α is enabled by its broad binding capacity to transcription factors in various signaling pathways. PGC-1α has multiple binding sites for the interactions with diverse coactivators. A domain between amino acids 200 and 400 interacts with the nuclear receptors PPARγ and NRF-1 [124], which are considered as master regulators of mitochondrial biogenesis [125]. PGC-1α binds to and activates the transcriptional function of NRF-1 on the promoter for TFAM, a direct regulator of mitochondrial DNA replication and transcription [120]. Another domain that predominantly binds to nuclear hormone receptors such as ERR-α, PPARs, RXR, glucocorticoid receptor, HNF4, and probably others, is an LXXLL sequence in the N-terminal region of PGC-1α [124]. This sequence is necessary for the coactivation of the nuclear receptor liver x receptor α (LXRα) [126]. The transcription complex of LXRα and PGC-1α then activates fatty acid synthase (FAS), a multifunctional enzyme that catalyzes all reactions required for the *de novo* biosynthesis of lipid [127]. The binding site of the nuclear receptor estrogen-related receptor α (ERR-α) is also in the LXXLL region of PGC-1α [124]. The transcription complex formed by ERR-α and PGC-1α induces the expression of vascular endothelial growth factor (VEGF), a potent stimulator of angiogenesis [128,129]. Between amino acids 400 to 500 of the PGC-1α protein is the binding site for myocyte enhancer factor 2 (MEF2). This transcription factor is a key regulator of slow muscle identity [130]. MEF2 proteins are activated through the calcium-regulated calcineurin signaling pathway [130,131]. When overexpressed, MEF2C promotes the formation of slow fibers, thus enhancing running endurance in mice [132]. Genetic deletion of *Mef2c* has been shown to block activity-dependent (exercise-induced) fast-to-slow fiber type transition [132]. This is in line with the proposed role of PGC-1α in such transitions. Muscle-specific overexpression of PGC-1α has been shown to evoke a transition of glycolytic type II in oxidative type I fibers [122]. This shift is initiated by the formation of a PGC-1α/MEF2 transcription complex, which then activates the expression of slow muscle genes [133]. Handschin *et al.* [123] have shown that PGC-1α deficient mice display a significant shift from slow oxidative type I and fast oxidative IIA toward fast glycolytic type IIX and IIB fibers, resulting in a reduced endurance capacity.

### 5.2. PGC-1α Regulates ROS Defense

It has been shown that oxidative stress increases the expression of PGC-1α [134]. Similarly, depleting the endogenous antioxidant glutathione augments exercise-mediated induction of PGC-1α expression [135]. Upregulation of PGC-1α possibly involves the transcription factor Cre-binding protein (CREB) [136]. PGC-1α then induces an increase of ROS-detoxifying enzymes, including GPX1 and Mn-SOD [136]. Only recently, new light has been shed on the molecular mechanisms involved in antioxidant activation. Therefore, it is highly likely that PGC-1α binds to ERR-α and activates the NAD^+^-dependent histone deacetylase silent information regulator 3 (SIRT3) in the mitochondrial matrix [137]. SIRT3 is also known to regulate ROS production by directly binding and deacetylating mitochondrial complex I and II [138,139]. Previous studies have also shown that SIRT3 is able to deacetylate the mitochondrial enzyme Mn-SOD, thereby promoting its antioxidative activity [140,141,142]. Thus, it appears that PGC-1α is a powerful suppressor of ROS production mainly by upregulation of antioxidant expression. Correspondingly, it was demonstrated that PGC-1α knock-out (KO) mice have reduced expression levels of Mn-SOD, Cu, Zn-SOD and GPX1 and are thus more sensitive to oxidative stressors [136]. By contrast, overexpression of PGC-1α enhances antioxidant defense by upregulation of Mn-SOD expression and a higher catalase activity [143]. Further, PGC-1α increases the expression of uncoupling proteins 2 and 3 (UCP2, UCP3) and thereby concomitantly reduces mitochondrial ROS production [144].

## 6. Conclusions

There is rapidly growing evidence that ROS have both positive and negative effects in contracting skeletal muscle cells. The deleterious effects such as a reduction of force generation and increased muscle atrophy appear to occur particularly after non-regular strenuous exercise, while regular training has positive effects by influencing cellular processes that lead to increased expression of antioxidants. These molecules then provide a better protection from ROS during subsequent trainings. However, a diet supplemented with exogenous antioxidants such as vitamins appears to prevent health-promoting effects of physical exercise in humans. The exercise-induced production of ROS may also be an important signal to activate PGC-1α, a key player in the adaption of muscle cells to exercise.

## Abbreviations

AMPK: adenosine monophosphate-activated protein kinase
ARE: antioxidant-responsive element
CAT: catalase
CHOP: C/EBP homology protein
CREB: Cre-binding protein
Cu, Zn-SOD: copper-zinc superoxide dismutase
eNOS: endothelial nitric oxide synthase
ERR-α: estrogen-related receptor α
FAS: fatty acid synthase
GPX: glutathione peroxidase
GR: glutathione reductase
GSH: glutathione
GSSG: glutathione disulfide
HNE: 4-hydroxynonenal
IFN-γ: interferon-γ
IL-1: interleukin-1
iNOS: inducible nitric oxide synthase
LXRα: nuclear receptor liver x receptor α
MAFbx: muscle atrophy F-box
MAPK: mitogen-activated protein kinase
MEF2: myocyte enhancer factor 2
Mn-SOD: manganese superoxide dismutase
MuRF-1: muscle RING-finger protein 1
NADPH: nicotinamide adenine dinucleotide phosphate
NF-κB: nuclear factor-κB
NOX: nicotinamide adenine dinucleotide phosphate oxidase
NRF: nuclear respiratory factor
Nrf2: nuclear factor erythroid-derived 2-like 2
OT: overloaded training
PGC-1α: peroxisome proliferator-activated receptor-γ coactivator-1α
PLA2: phospholipase A2
PPARγ: peroxisome proliferator-activated receptor γ
ROS: reactive oxygen species
RyR: ryanodine receptor
SIRT3: silent information regulator 3
TBARS: thiobarbituric acid reactive substance
TFAM: mitochondrial transcription factor A
TNF: tumor necrosis factor
TxnRd2,: thioredoxin reductase-2
UCP: uncoupling protein
VEGF: vascular endothelial growth factor
XO: xanthine oxidase

## References

[1] Dillard C.J., Litov R.E., Savin W.M., Dumelin E.E., Tappel A.L. (1978). Effects of exercise, vitamin E, and ozone on pulmonary function and lipid peroxidation. J. Appl. Physiol. Respir. Environ. Exerc. Physiol..

[2] Davies K.J., Quintanilha A.T., Brooks G.A., Packer L. (1982). Free radicals and tissue damage produced by exercise. Biochem. Biophys. Res. Commun..

[3] Vollaard N.B., Shearman J.P., Cooper C.E. (2005). Exercise-induced oxidative stress: Myths, realities and physiological relevance. Sports Med..

[4] Fisher-Wellman K., Bloomer R.J. (2009). Acute exercise and oxidative stress: A 30 year history. Dyn. Med..

[5] Sahlin K., Shabalina I.G., Mattsson C.M., Bakkman L., Fernström M., Rozhdestvenskaya Z., Enqvist J.K., Nedergaard J., Ekblom B., Tonkonogi M. (2010). Ultraendurance exercise increases the production of reactive oxygen species in isolated mitochondria from human skeletal muscle. J. Appl. Physiol..

[6] Hey-Mogensen M., Højlund K., Vind B.F., Wang L., Dela F., Beck-Nielsen H., Fernström M., Sahlin K. (2010). Effect of physical training on mitochondrial respiration and reactive oxygen species release in skeletal muscle in patients with obesity and type 2 diabetes. Diabetologia.

[7] Ghosh S., Lertwattanarak R., Lefort N., Molina-Carrion M., Joya-Galeana J., Bowen B.P., Garduno-Garcia Jde J., Abdul-Ghani M., Richardson A., DeFronzo R.A. (2011). Reduction in reactive oxygen species production by mitochondria from elderly subjects with normal and impaired glucose tolerance. Diabetes.

[8] Powers S.K., Jackson M.J. (2008). Exercise-induced oxidative stress: Cellular mechanisms and impact on muscle force production. Physiol. Rev..

[9] Peake J., Suzuki K. (2004). Neutrophil activation, antioxidant supplements and exercise-induced oxidative stress. Exerc. Immunol. Rev..

[10] Moylan J.S., Reid M.B. (2007). Oxidative stress, chronic disease, and muscle wasting. Muscle Nerve.

[11] Gomes E.C., Silva A.N., de Oliveira M.R. (2012). Oxidants, antioxidants, and the beneficial roles of exercise-induced production of reactive species. Oxid. Med. Cell Longev..

[12] Duarte J.A., Appell H.J., Carvalho F., Bastos M.L., Soares J.M. (1993). Endothelium-derived oxidative stress may contribute to exercise-induced muscle damage. Int. J. Sports Med..

[13] Murphy M.P. (2009). How mitochondria produce reactive oxygen species. Biochem. J..

[14] Brand M.D. (2010). The sites and topology of mitochondrial superoxide production. Exp. Gerontol..

[15] St-Pierre J., Buckingham J.A., Roebuck S.J., Brand M.D. (2002). Topology of superoxide production from different sites in the mitochondrial electron transport chain. J. Biol. Chem..

[16] Perevoshchikova I.V., Quinlan C.L., Orr A.L., Gerencser A.A., Brand M.D. (2013). Sites of superoxide and hydrogen peroxide production during fatty acid oxidation in rat skeletal muscle mitochondria. Free Radic. Biol. Med..

[17] Hey-Mogensen M., Goncalves R.L., Orr A.L., Brand M.D. (2014). Production of superoxide/H_2_O_2_ by dihydroorotate dehydrogenase in rat skeletal muscle mitochondria. Free Radic. Biol. Med..

[18] Barja G. (1999). Mitochondrial oxygen radical generation and leak: Sites of production in states 4 and 3, organ specificity, and relation to aging and longevity. J. Bioenerg. Biomembr..

[19] Muller F.L., Liu Y., van Remmen H. (2004). Complex III releases superoxide to both sides of the inner mitochondrial membrane. J. Biol. Chem..

[20] Goncalves R.L., Quinlan C.L., Perevoshchikova I.V., Hey-Mogensen M., Brand M.D. (2015). Sites of superoxide and hydrogen peroxide production by muscle mitochondria assessed *ex vivo* under conditions mimicking rest and exercise. J. Biol. Chem..

[21] Bedard K., Krause K.H. (2007). The NOX family of ROS-generating NADPH oxidases: Physiology and pathophysiology. Physiol. Rev..

[22] Brandes R.P., Weissmann N., Schröder K. (2014). Nox family NADPH oxidases: Molecular mechanisms of activation. Free Radic. Biol. Med..

[23] Xia R., Webb J.A., Gnall L.L., Cutler K., Abramson J.J. (2003). Skeletal muscle sarcoplasmic reticulum contains a NADH-dependent oxidase that generates superoxide. Am. J. Physiol. Cell Physiol..

[24] Shkryl V.M., Martins A.S., Ullrich N.D., Nowycky M.C., Niggli E., Shirokova N. (2009). Reciprocal amplification of ROS and Ca^2+^ signals in stressed mdx dystrophic skeletal muscle fibers. Pflugers Arch..

[25] Sakellariou G.K., Vasilaki A., Palomero J., Kayani A., Zibrik L., McArdle A., Jackson M.J. (2013). Studies of mitochondrial and nonmitochondrial sources implicate nicotinamide adenine dinucleotide phosphate oxidase(s) in the increased skeletal muscle superoxide generation that occurs during contractile activity. Antioxid. Redox Signal..

[26] Cherednichenko G., Zima A.V., Feng W., Schaefer S., Blatter L.A., Pessah I.N. (2004). NADH oxidase activity of rat cardiac sarcoplasmic reticulum regulates calcium-induced calcium release. Circ. Res..

[27] Espinosa A., Leiva A., Peña M., Müller M., Debandi A., Hidalgo C., Carrasco M.A., Jaimovich E. (2006). Myotube depolarization generates reactive oxygen species through NAD(P)H oxidase; ROS-elicited Ca^2+^ stimulates ERK, CREB, early genes. J. Cell Physiol..

[28] Hidalgo C., Sánchez G., Barrientos G., Aracena-Parks P. (2006). A transverse tubule NADPH oxidase activity stimulates calcium release from isolated triads via ryanodine receptor type 1 *S*-glutathionylation. J. Biol. Chem..

[29] Espinosa A., García A., Härtel S., Hidalgo C., Jaimovich E. (2009). NADPH oxidase and hydrogen peroxide mediate insulin-induced calcium increase in skeletal muscle cells. J. Biol. Chem..

[30] Harrison R. (2002). Structure and function of xanthine oxidoreductase: Where are we now?. Free Radic. Biol. Med..

[31] Judge A.R., Dodd S.L. (2004). Xanthine oxidase and activated neutrophils cause oxidative damage to skeletal muscle after contractile claudication. Am. J. Physiol. Heart Circ. Physiol..

[32] Radák Z., Zhao Z., Koltai E., Ohno H., Atalay M. (2013). Oxygen consumption and usage during physical exercise: The balance between oxidative stress and ROS-dependent adaptive signaling. Antioxid. Redox Signal..

[33] Kang C., Chung E., Diffee G., Ji L.L. (2013). Exercise training attenuates aging-associated mitochondrial dysfunction in rat skeletal muscle: Role of PGC-1α. Exp. Gerontol..

[34] Sriram S., Subramanian S., Sathiakumar D., Venkatesh R., Salerno M.S., McFarlane C.D., Kambadur R., Sharma M. (2011). Modulation of reactive oxygen species in skeletal muscle by myostatin is mediated through NF-κB. Aging Cell.

[35] Sriram S., Subramanian S., Juvvuna P.K., Ge X., Lokireddy S., McFarlane C.D., Wahli W., Kambadur R., Sharma M. (2014). Myostatin augments muscle-specific ring finger protein-1 expression through an NF-κB independent mechanism in SMAD3 null muscle. Mol. Endocrinol..

[36] Zuo L., Christofi F.L., Wright V.P., Bao S., Clanton T.L. (2004). Lipoxygenase-dependent superoxide release in skeletal muscle. J. Appl. Physiol..

[37] Zhao X., Bey E.A., Wientjes F.B., Cathcart M.K. (2002). Cytosolic phospholipase A2 (cPLA2) regulation of human monocyte NADPH oxidase activity. cPLA2 affects translocation but not phosphorylation of p67(phox) and p47(phox). J. Biol. Chem..

[38] Ježek J., Jaburek M., Zelenka J., Ježek P. (2010). Mitochondrial phospholipase A2 activated by reactive oxygen species in heart mitochondria induces mild uncoupling. Physiol. Res..

[39] Gong M.C., Arbogast S., Guo Z., Mathenia J., Su W., Reid M.B. (2006). Calcium-independent phospholipase A2 modulates cytosolic oxidant activity and contractile function in murine skeletal muscle cells. J. Appl. Physiol..

[40] Smith M.A., Reid M.B. (2006). Redox modulation of contractile function in respiratory and limb skeletal muscle. Respir. Physiol. Neurobiol..

[41] Radák Z., Taylor A.W., Ohno H., Goto S. (2001). Adaptation to exercise-induced oxidative stress: From muscle to brain. Exerc. Immunol. Rev..

[42] Reid M.B. (2008). Free radicals and muscle fatigue: Of ROS, canaries, and the IOC. Free Radic. Biol. Med..

[43] Allen D.G., Lamb G.D., Westerblad H. (2008). Skeletal muscle fatigue: Cellular mechanisms. Physiol. Rev..

[44] Lamb G.D., Westerblad H. (2011). Acute effects of reactive oxygen and nitrogen species on the contractile function of skeletal muscle. J. Physiol..

[45] Lamb G.D., Posterino G.S. (2003). Effects of oxidation and reduction on contractile function in skeletal muscle fibres of the rat. J. Physiol..

[46] Posterino G.S., Cellini M.A., Lamb G.D. (2003). Effects of oxidation and cytosolic redox conditions on excitation-contraction coupling in rat skeletal muscle. J. Physiol..

[47] Cheng A.J., Bruton J.D., Lanner J.T., Westerblad H. (2015). Antioxidant treatments do not improve force recovery after fatiguing stimulation of mouse skeletal muscle fibres. J. Physiol..

[48] Andrade F.H., Reid M.B., Allen D.G., Westerblad H. (1998). Effect of hydrogen peroxide and dithiothreitol on contractile function of single skeletal muscle fibres from the mouse. J. Physiol..

[49] Prochniewicz E., Spakowicz D., Thomas D.D. (2008). Changes in actin structural transitions associated with oxidative inhibition of muscle contraction. Biochemistry.

[50] Mollica J.P., Dutka T.L., Merry T.L., Lamboley C.R., McConell G.K., McKenna M.J., Murphy R.M., Lamb G.D. (2012). *S*-glutathionylation of troponin I (fast) increases contractile apparatus Ca^2+^ sensitivity in fast-twitch muscle fibres of rats and humans. J. Physiol..

[51] Jackson M.J., Papa S., Bolaños J., Bruckdorfer R., Carlsen H., Elliott R.M., Flier J., Griffiths H.R., Heales S., Holst B. (2002). Antioxidants, reactive oxygen and nitrogen species, gene induction and mitochondrial function. Mol. Aspects Med..

[52] Gumucio J.P., Mendias C.L. (2013). Atrogin-1, MuRF-1, and sarcopenia. Endocrine.

[53] Witt S.H., Granzier H., Witt C.C., Labeit S. (2005). MURF-1 and MURF-2 target a specific subset of myofibrillar proteins redundantly: Towards understanding MURF-dependent muscle ubiquitination. J. Mol. Biol..

[54] Cohen S., Brault J.J., Gygi S.P., Glass D.J., Valenzuela D.M., Gartner C., Latres E., Goldberg A.L. (2009). During muscle atrophy, thick, but not thin, filament components are degraded by MuRF1-dependent ubiquitylation. J. Cell Biol..

[55] Ji L.L. (1999). Antioxidants and oxidative stress in exercise. Proc. Soc. Exp. Biol. Med..

[56] Smith C.V., Jones D.P., Guenther T.M., Lash L.H., Lauterburg B.H. (1996). Compartmentation of glutathione: Implications for the study of toxicity and disease. Toxicol. Appl. Pharmacol..

[57] Hayes J.D., Flanagan J.U., Jowsey I.R. (2005). Glutathione transferases. Ann. Rev. Pharmacol. Toxicol..

[58] Meister A., Anderson M.E. (1983). Glutathione. Annu. Rev. Biochem..

[59] Halliwell B., Gutteridge J. (2007). Free Radicals in Biology and Medicine.

[60] Bellomo G., Mirabelli F., DiMonte D., Richelmi P., Thor H., Orrenius C., Orrenius S. (1987). Formation and reduction of glutathione-protein mixed disulfides during oxidative stress. A study with isolated hepatocytes and menadione (2-methyl-1,4-naphthoquinone). Biochem. Pharmacol..

[61] Ji L.L., Fu R., Mitchell E.W. (1992). Glutathione and antioxidant enzymes in skeletal muscle: Effects of fiber type and exercise intensity. J. Appl. Physiol..

[62] Lew H., Pyke S., Quintanilha A. (1985). Changes in the glutathione status of plasma, liver and muscle following exhaustive exercise in rats. FEBS Lett..

[63] Marin E., Kretzschmar M., Arokoski J., Hänninen O., Klinger W. (1993). Enzymes of glutathione synthesis in dog skeletal muscles and their response to training. Acta Physiol. Scand..

[64] Leeuwenburgh C., Hollander J., Leichtweis S., Griffiths M., Gore M., Ji L.L. (1997). Adaptations of glutathione antioxidant system to endurance training are tissue and muscle fiber specific. Am. J. Physiol..

[65] Ji L.L. (1995). Exercise and oxidative stress: Role of the cellular antioxidant systems. Exerc. Sport Sci. Rev..

[66] Sen C.K. (1995). Oxidants and antioxidants in exercise. J. Appl. Physiol..

[67] Fisher-Wellman K.H., Mattox T.A., Thayne K., Katunga L.A., la Favor J.D., Neufer P.D., Hickner R.C., Wingard C.J., Anderson E.J. (2013). Novel role for thioredoxin reductase-2 in mitochondrial redox adaptations to obesogenic diet and exercise in heart and skeletal muscle. J. Physiol..

[68] Ji L.L. (2008). Modulation of skeletal muscle antioxidant defense by exercise: Role of redox signaling. Free Radic. Biol. Med..

[69] Powers S.K., Criswell D., Lawler J., Martin D., Ji L.L., Herb R.A., Dudley G. (1994). Regional training-induced alterations in diaphragmatic oxidative and antioxidant enzymes. Respir. Physiol..

[70] Gore M., Fiebig R., Hollander J., Leeuwenburgh C., Ohno H., Ji L.L. (1998). Endurance training alters antioxidant enzyme gene expression in rat skeletal muscle. Can. J. Physiol. Pharmacol..

[71] Hollander J., Fiebig R., Gore M., Bejma J., Ookawara T., Ohno H., Ji L.L. (1999). Superoxide dismutase gene expression in skeletal muscle: Fiber-specific adaptation to endurance training. Am. J. Physiol..

[72] Lambertucci R.H., Levada-Pires A.C., Rossoni L.V., Curi R., Pithon-Curi T.C. (2007). Effects of aerobic exercise training on antioxidant enzyme activities and mRNA levels in soleus muscle from young and aged rats. Mech. Ageing Dev..

[73] Ristow M., Zarse K., Oberbach A., Klöting N., Birringer M., Kiehntopf M., Stumvoll M., Kahn C.R., Blüher M. (2009). Antioxidants prevent health-promoting effects of physical exercise in humans. Proc. Natl. Acad. Sci. USA.

[74] Ji L.L., Fu R. (1992). Responses of glutathione system and antioxidant enzymes to exhaustive exercise and hydroperoxide. J. Appl. Physiol..

[75] Lawler J.M., Powers S.K., Visser T., van Dijk H., Kordus M.J., Ji L.L. (1993). Acute exercise and skeletal muscle antioxidant and metabolic enzymes: Effects of fiber type and age. Am. J. Physiol..

[76] Hollander J., Fiebig R., Gore M., Ookawara T., Ohno H., Ji L.L. (2001). Superoxide dismutase gene expression is activated by a single bout of exercise in rat skeletal muscle. Pflugers Arch..

[77] Hitomi Y., Watanabe S., Kizaki T., Sakurai T., Takemasa T., Haga S., Ookawara T., Suzuki K., Ohno H. (2008). Acute exercise increases expression of extracellular superoxide dismutase in skeletal muscle and the aorta. Redox. Rep..

[78] Radák Z., Asano K., Inoue M., Kizaki T., Oh-Ishi S., Suzuki K., Taniguchi N., Ohno H. (1995). Superoxide dismutase derivative reduces oxidative damage in skeletal muscle of rats during exhaustive exercise. J. Appl. Physiol..

[79] Alessio H.M., Goldfarb A.H. (1988). Lipid peroxidation and scavenger enzymes during exercise: Adaptive response to training. J. Appl. Physiol..

[80] Mastaloudis A., Leonard S.W., Traber M.G. (2001). Oxidative stress in athletes during extreme endurance exercise. Free Radic. Biol. Med..

[81] Steensberg A., Morrow J., Toft A.D., Bruunsgaard H., Pedersen B.K. (2002). Prolonged exercise, lymphocyte apoptosis and F2-isoprostanes. Eur. J. Appl. Physiol..

[82] Ashton T., Rowlands C.C., Jones E., Young I.S., Jackson S.K., Davies B., Peters J.R. (1998). Electron spin resonance spectroscopic detection of oxygen-centred radicals in human serum following exhaustive exercise. Eur. J. Appl. Physiol. Occup. Physiol..

[83] Vasankari T., Kujala U., Heinonen O., Kapanen J., Ahotupa M. (1995). Measurement of serum lipid peroxidation during exercise using three different methods: Diene conjugation, thiobarbituric acid reactive material and fluorescent chromolipids. Clin. Chim. Acta.

[84] Umegaki K., Daohua P., Sugisawa A., Kimura M., Higuchi M. (2000). Influence of one bout of vigorous exercise on ascorbic acid in plasma and oxidative damage to DNA in blood cells and muscle in untrained rats. J. Nutr. Biochem..

[85] Niess A.M., Hartmann A., Grünert-Fuchs M., Poch B., Speit G. (1996). DNA damage after exhaustive treadmill running in trained and untrained men. Int. J. Sports Med..

[86] Powers S.K., Criswell D., Lawler J., Ji L.L., Martin D., Herb R.A., Dudley G. (1994). Influence of exercise and fiber type on antioxidant enzyme activity in rat skeletal muscle. Am. J. Physiol..

[87] Radák Z., Naito H., Kaneko T., Tahara S., Nakamoto H., Takahashi R., Cardozo-Pelaez F., Goto S. (2002). Exercise training decreases DNA damage and increases DNA repair and resistance against oxidative stress of proteins in aged rat skeletal muscle. Pflugers Arch..

[88] Radák Z., Chung H.Y., Goto S. (2008). Systemic adaptation to oxidative challenge induced by regular exercise. Free Radic. Biol. Med..

[89] Alleman R.J., Katunga L.A., Nelson M.A.M., Brown D.A., Anderson E.J. (2014). The “Goldilocks Zone” from a redox perspective—Adaptive *vs.* deleterious responses to oxidative stress in striated muscle. Front. Physiol..

[90] Kalogeris T., Bao Y., Korthuis R.J. (2014). Mitochondrial reactive oxygen species: A double edged sword in ischemia/reperfusion *vs.* preconditioning. Redox Biol..

[91] Dodd S.L., Gagnon B.J., Senf S.M., Hain B.A., Judge A.R. (2010). Ros-mediated activation of NF-kappaB and Foxo during muscle disuse. Muscle Nerve.

[92] Derbre F., Ferrando B., Gomez-Cabrera M.C., Sanchis-Gomar F., Martinez-Bello V.E., Olaso-Gonzalez G., Diaz A., Gratas-Delamarche A., Cerda M., Viña J. (2012). Inhibition of xanthine oxidase by allopurinol prevents skeletal muscle atrophy: Role of p38 MAPKinase and E3 ubiquitin ligases. PLoS ONE.

[93] Kröller-Schön S., Jansen T., Hauptmann F., Schüler A., Heeren T., Hausding M., Oelze M., Viollet B., Keaney J.F., Wenzel P. (2012). α1AMP-activated protein kinase mediates vascular protective effects of exercise. Arterioscler. Thromb. Vasc. Biol..

[94] Morgan M.J., Liu Z.G. (2011). Crosstalk of reactive oxygen species and NF-κB signaling. Cell Res..

[95] McArdle F., Spiers S., Aldemir H., Vasilaki A., Beaver A., Iwanejko L., McArdle A., Jackson M.J. (2004). Preconditioning of skeletal muscle against contraction-induced damage: The role of adaptations to oxidants in mice. J. Physiol..

[96] Eckl P.M., Ortner A., Esterbauer H. (1993). Genotoxic properties of 4-hydroxyalkenals and analogous aldehydes. Mutat. Res..

[97] Poli G., Schaur R.J., Siems W.G., Leonarduzzi G. (2008). 4-hydroxynonenal: A membrane lipid oxidation product of medicinal interest. Med. Res. Rev..

[98] Ishii T., Itoh K., Ruiz E., Leake D.S., Unoki H., Yamamoto M., Mann G.E. (2004). Role of Nrf2 in the regulation of CD36 and stress protein expression in murine macrophages: Activation by oxidatively modified LDL and 4-hydroxynonenal. Circ. Res..

[99] Levonen A.L., Landar A., Ramachandra A., Cease E.K., Dickinson D.A., Zanoni G., Morrow J.D., Darley-Usmar V.M. (2004). Cellular mechanisms of redox cell signalling: Role of cysteine modification in controlling antioxidant defences in response to electrophilic lipid oxidation products. Biochem. J..

[100] Echtay K.S., Esteves T.C., Pakay J.L., Jekabsons M.B., Lambert A.J., Portero-Otín M., Pamplona R., Vidal-Puig A.J., Wang S., Roebuck S.J. (2003). A signalling role for 4-hydroxy-2-nonenal in regulation of mitochondrial uncoupling. EMBO J..

[101] Gomez-Cabrera M.C., Borrás C., Pallardó F.V., Sastre J., Ji L.L., Viña J. (2005). Decreasing xanthine oxidase-mediated oxidative stress prevents useful cellular adaptations to exercise in rats. J. Physiol..

[102] Gomez-Cabrera M.C., Domenech E., Viña J. (2008). Moderate exercise is an antioxidant: Upregulation of antioxidant genes by training. Free Radic. Biol. Med..

[103] Ji L.L., Gomez-Cabrera M.C., Steinhafel N., Viña J. (2004). Acute exercise activates nuclear factor (NF)-kappaB signaling pathway in rat skeletal muscle. FASEB J..

[104] Margonis K., Fatouros I.G., Jamurtas A.Z., Nikolaidis M.G., Douroudos I., Chatzinikolaou A., Mitrakou A., Mastorakos G., Papassotiriou I., Taxildaris K. (2007). Oxidative stress biomarkers responses to physical overtraining: Implications for diagnosis. Free Radic. Biol. Med..

[105] Palazzetti S., Richard M.J., Favier A., Margaritis I. (2003). Overloaded training increases exercise-induced oxidative stress and damage. Can. J. Appl. Physiol..

[106] Tanskanen M., Atalay M., Uusitalo A. (2010). Altered oxidative stress in overtrained athletes. J. Sports Sci..

[107] Irrcher I., Adhihetty P.J., Sheehan T., Joseph A.M., Hood D.A. (2003). PPARγ coactivator-1α expression during thyroid hormone- and contractile activity-induced mitochondrial adaptations. Am. J. Physiol. Cell Physiol..

[108] Holloszy J.O. (2008). Regulation by exercise of skeletal muscle content of mitochondria and GLUT4. J. Physiol. Pharmacol..

[109] Yan Z., Okutsu M., Akhtar Y.N., Lira V.A. (2011). Regulation of exercise-induced fiber type transformation, mitochondrial biogenesis, and angiogenesis in skeletal muscle. J. Appl. Physiol..

[110] Van Loon L.J.C., Goodpaster B.H. (2006). Increased intramuscular lipid storage in the insulin-resistant and endurance-trained state. Pflugers Arch..

[111] Hawley J.A., Lessard S.J. (2008). Exercise training-induced improvements in insulin action. Acta Physiol..

[112] Olesen J., Kiilerich K., Pilegaard H. (2010). PGC-1α-mediated adaptations in skeletal muscle. Pflugers Arch..

[113] Kang C., Ji L.L. (2013). Role of PGC-1α in muscle function and aging. J. Sport Health Sci..

[114] Pilegaard H., Osada T., Andersen L.T., Helge J.W., Saltin B., Neufer P.D. (2005). Substrate availability and transcriptional regulation of metabolic genes in human skeletal muscle during recovery from exercise. Metabolism.

[115] Krämer D.K., Ahlsen M., Norrbom J., Jansson E., Hjeltnes N., Gustafsson T., Krook A. (2006). Human skeletal muscle fibre type variations correlate with PPARα, PPARδ and PGC-1α mRNA. Acta Physiol..

[116] Mathai A.S., Bonen A., Benton C.R., Robinson D.L., Graham T.E. (2008). Rapid exercise-induced changes in PGC-1α mRNA and protein in human skeletal muscle. J. Appl. Physiol..

[117] Little J.P., Safdar A., Bishop D., Tarnopolsky M.A., Gibala M.J. (2011). An acute bout of high-intensity interval training increases the nuclear abundance of PGC-1α and activates mitochondrial biogenesis in human skeletal muscle. Am. J. Physiol. Regul. Integr. Comp. Physiol..

[118] Cobley J.N., Bartlett J.D., Kayani A., Murray S.W., Louhelainen J., Donovan T., Waldron S., Gregson W., Burniston J.G., Morton J.P. (2012). PGC-1α transcriptional response and mitochondrial adaptation to acute exercise is maintained in skeletal muscle of sedentary elderly males. Biogerontology.

[119] Wright D.C., Han D.H., Garcia-Roves P.M., Geiger P.C., Jones T.E., Holloszy J. (2007). Exercise-induced mitochondrial biogenesis begins before the increase in muscle PGC-1α expression. J. Biol. Chem..

[120] Wu Z., Puigserver P., Andersson U., Zhang C., Adelmant G., Mootha V., Troy A., Cinti S., Lowell B., Scarpulla R.C. (1999). Mechanisms controlling mitochondrial biogenesis and respiration through the thermogenic coactivator PGC-1. Cell.

[121] Koves T.R., Li P., An J., Akimoto T., Slentz D., Ilkayeva O., Dohm G.L., Yan Z., Newgard C.B., Muoio D.M. (2005). Peroxisome proliferator-activated receptor-γ co-activator 1α-mediated metabolic remodeling of skeletal myocytes mimics exercise training and reverses lipid-induced mitochondrial inefficiency. J. Biol. Chem..

[122] Lin J., Wu H., Tarr P.T., Zhang C.Y., Wu Z., Boss O., Michael L.F., Puigserver P., Isotani E., Olson E.N. (2002). Transcriptional co-activator PGC-1α drives the formation of slow-twitch muscle fibres. Nature.

[123] Handschin C., Chin S., Li P., Liu F., Maratos-Flier E., Lebrasseur N.K., Yan Z., Spiegelman B.M. (2007). Skeletal muscle fiber-type switching, exercise intolerance, and myopathy in PGC-1α muscle-specific knock-out animals. J. Biol. Chem..

[124] Puigserver P., Spiegelman B.M. (2003). Peroxisome proliferator-activated receptor-γ coactivator 1α (PGC-1α): Transcriptional coactivator and metabolic regulator. Endocr. Rev..

[125] Scarpulla R.C., Vega R.B., Kelly D.P. (2012). Transcriptional integration of mitochondrial biogenesis. Trends Endocrinol. Metab..

[126] Oberkofler H., Schraml E., Krempler F., Patsch W. (2003). Potentiation of liver X receptor transcriptional activity by peroxisome-proliferator-activated receptor γ co-activator 1α. Biochem. J..

[127] Summermatter S., Baum O., Santos G., Hoppeler H., Handschin C. (2010). Peroxisome proliferator-activated receptor γ coactivator 1α (PGC-1α) promotes skeletal muscle lipid refueling *in vivo* by activating *de novo* lipogenesis and the pentose phosphate pathway. J. Biol. Chem..

[128] Arany Z., Foo S.Y., Ma Y., Ruas J.L., Bommi-Reddy A., Girnun G., Cooper M., Laznik D., Chinsomboon J., Rangwala S.M. (2008). HIF-independent regulation of VEGF and angiogenesis by the transcriptional coactivator PGC-1α. Nature.

[129] Chinsomboon J., Ruas J., Gupta R.K., Thom R., Shoag J., Rowe G.C., Sawada N., Raghuram S., Arany Z. (2009). The transcriptional coactivator PGC-1α mediates exercise-induced angiogenesis in skeletal muscle. Proc. Natl. Acad. Sci. USA.

[130] Chin E.R., Olson E.N., Richardson J.A., Yang Q., Humphries C., Shelton J.M., Wu H., Zhu W., Bassel-Duby R., Williams R.S. (1998). A calcineurin-dependent transcriptional pathway controls skeletal muscle fiber type. Genes Dev..

[131] Wu H., Naya F.J., McKinsey T.A., Mercer B., Shelton J.M., Chin E.R., Simard A.R., Michel R.N., Bassel-Duby R., Olson E.N. (2000). MEF2 responds to multiple calcium-regulated signals in the control of skeletal muscle fiber type. EMBO J..

[132] Potthoff M.J., Wu H., Arnold M.A., Shelton J.M., Backs J., McAnally J., Richardson J.A., Bassel-Duby R., Olson E.N. (2007). Histone deacetylase degradation and MEF2 activation promote the formation of slow-twitch myofibers. J. Clin. Investig..

[133] Handschin C., Rhee J., Lin J., Tarr P.T., Spiegelman B.M. (2003). An autoregulatory loop controls peroxisome proliferator-activated receptor γ coactivator 1α expression in muscle. Proc. Natl. Acad. Sci. USA.

[134] Kang C., O’Moore K.M., Dickman J.R., Ji L.L. (2009). Exercise activation of muscle peroxisome proliferator-activated receptor-gamma coactivator-1α signaling is redox sensitive. Free Radic. Biol. Med..

[135] Strobel N.A., Matsumoto A., Peake J.M., Marsh S.A., Peternelj T.T., Briskey D., Fassett R.G., Coombes J.S., Wadley G.D. (2014). Altering the redox state of skeletal muscle by glutathione depletion increases the exercise-activation of PGC-1α. Physiol. Rep..

[136] St-Pierre J., Drori S., Uldry M., Silvaggi J.M., Rhee J., Jäger S., Handschin C., Zheng K., Lin J., Yang W. (2006). Suppression of reactive oxygen species and neurodegeneration by the PGC-1 transcriptional coactivators. Cell.

[137] Kong X., Wang R., Xue Y., Liu X., Zhang H., Chen Y., Fang F., Chang Y. (2010). Sirtuin 3, a new target of PGC-1α, plays an important role in the suppression of ROS and mitochondrial biogenesis. PLoS ONE.

[138] Ahn B.H., Kim H.S., Song S., Lee I.H., Liu J., Vassilopoulos A., Deng C.X., Finkel T. (2008). A role for the mitochondrial deacetylase Sirt3 in regulating energy homeostasis. Proc. Natl. Acad. Sci. USA.

[139] Finley L.W., Haas W., Desquiret-Dumas V., Wallace D.C., Procaccio V., Gygi S.P., Haigis M.C. (2011). Succinate dehydrogenase is a direct target of sirtuin 3 deacetylase activity. PLoS ONE.

[140] Qiu X., Brown K., Hirschey M.D., Verdin E., Chen D. (2010). Calorie restriction reduces oxidative stress by SIRT3-mediated SOD2 activation. Cell Metab..

[141] Tao R., Coleman M.C., Pennington J.D., Ozden O., Park S.H., Jiang H., Kim H.S., Flynn C.R., Hill S., Hayes McDonald W. (2010). Sirt3-mediated deacetylation of evolutionarily conserved lysine 122 regulates MnSOD activity in response to stress. Mol. Cell.

[142] Chen Y., Zhang J., Lin Y., Lei Q., Guan K.L., Zhao S., Xiong Y. (2011). Tumour suppressor SIRT3 deacetylates and activates manganese superoxide dismutase to scavenge ROS. EMBO Rep..

[143] Wenz T., Rossi S.G., Rotundo R.L., Spiegelman B.M., Moraes C.T. (2009). Increased muscle PGC-1α expression protects from sarcopenia and metabolic disease during aging. Proc. Natl. Acad. Sci. USA.

[144] St-Pierre J., Lin J., Krauss S., Tarr P.T., Yang R., Newgard C.B., Spiegelman B.M. (2003). Bioenergetic analysis of peroxisome proliferator-activated receptor γ coactivators 1α and 1β (PGC-1α and PGC-1β) in muscle cells. J. Biol. Chem..

